# Cardiac output monitoring: throw it out… or keep it?

**DOI:** 10.1186/s13054-018-1957-5

**Published:** 2018-02-08

**Authors:** Xavier Monnet, Jean-Louis Teboul

**Affiliations:** 1 Hôpitaux Universitaires Paris-Sud, Hôpital de Bicêtre, service de réanimation médicale, 78, rue du Général Leclerc, F-94270 Le Kremlin-Bicêtre, France; 2 Université Paris-Sud, Faculté de médecine Paris-Sud, EA4533, Le Kremlin-Bicêtre, F-94270 France

**Keywords:** Cardiac output, Haemodynamic monitoring, Thermodilution, Shock

## Background

In critical care units, the shelf for cardiac output (CO) monitoring devices fills up with ever more innovative systems. Are these techniques useful, or are they expensive and irrelevant gadgets? There are arguments to defend both viewpoints. In this commentary, which we have drawn up as a ‘pro–con debate’, we expose these arguments and deliver our opinion.

## Throw it out!

During circulatory failure, the variable that really matters is not CO but oxygen delivery and ultimately tissue oxygenation. However, a given value of CO does not necessarily inform on tissue oxygenation. Firstly, CO depends much on global oxygen demand; thus an apparently high CO may be inadequate (high oxygen demand), whereas a low CO might perfectly fit requirements (low oxygen demand). Secondly, CO is not the only determinant of oxygen delivery. For instance, even if fluid increases CO, the oxygen delivery to the tissues may increase to a smaller extent due to the inherent haemodilution [1]. Assessing global tissue oxygenation, through clinical examination, lactate, and central venous oxygenation (ScvO_2_), should be much more relevant than monitoring CO. Thirdly, tissue oxygen supply primarily depends on microcirculation. Under physiological conditions, CO and microcirculatory flows are coupled. However, in some circumstances, the most typical being sepsis, the regulation of microcirculatory flows is impaired and “coherence” between the macro- and microcirculation is lost [2]. In this regard, increasing CO cannot guarantee any parallel correction of microcirculatory abnormalities [3]. As an illustration, a clinical study showed that oxygen consumption improves in only half of patients in whom fluid infusion increases CO [1]. When deciding to continue fluid infusion or not, are not effects on tissue oxygenation more important than CO?

To justify its monitoring, CO should at least be a target for haemodynamic resuscitation which, unlike arterial pressure, is not actually the case. Also, the arterial pulse pressure is physiologically related to stroke volume. Rather than using costly and often invasive CO monitoring, could we not simply measure arterial pressure along with the above-mentioned tissue oxygenation variables?

Finally, studies repeatedly show that using CO monitoring devices during shock does not improve outcome [4]. All these arguments may discourage us from monitoring CO in critically ill patients…

## … but should we really?

The absence of any demonstrated benefit of haemodynamic monitoring is not a definitive argument. Nobody would administer norepinephrine without monitoring its effects on arterial pressure. Then, why should we use treatments aimed at increasing CO without directly checking their efficacy? Shock is so a complex disease that it is illusory that monitoring one single light on the dashboard can change prognosis. What may influence prognosis is not the monitoring, but the therapeutic decisions inferred from it. Odds are that this will never be properly demonstrated by any randomised trial, since a protocol taking into account all alternatives is impossible to establish.

Though oxygen delivery matters, CO is its main determinant and, often, the only lever one can operate to increase it. If CO should not be monitored on the pretext that only oxygen delivery matters, haemoglobin should not be measured after red blood cell transfusion.

Tailoring the treatment to the individual patient is a goal to reach, particularly for reducing harm due to therapy. Nevertheless, CO is highly variable depending on the type of shock, on the patient, and on the timing. For instance, the effects of volume expansion are not only variable among patients, but also transient [5]. This variability spurs CO measurement. Directly monitoring it is the unique way to do so since its clinical estimation is not reliable. In 530 patients, the presence of one clinical criterion among lengthening of capillary filling time, knees mottling, and cold extremities was totally inaccurate to detect a low CO [6]. Not only is there no “normal” value of CO, but also a high value of CO might co-exist with such signs of hypoperfusion.

Those who are reluctant to monitor CO often argue that monitoring arterial pressure is more than sufficient. Arterial pressure, which determines organ perfusion, is a relevant therapeutic target, but CO, which determines oxygen delivery, is no less vital. Moreover, when CO changes, the sympathetic tone adapts to keep the mean arterial pressure constant. Even arterial pulse pressure, which should be physiologically the closest CO correlate, is unhelpful. Studies have shown that during volume expansion changes in CO and in pulse pressure were weakly correlated [7] or not correlated at all [8]. This is particularly true when arterial resistance changes, for instance due to changing vasopressors dosage [7].

Finally, haemodynamic monitoring cannot be merely limited to tissue oxygenation and ScvO_2_. It is correct that CO cannot be interpreted without knowing it, but the opposite is also true. During sepsis, if oxygen extraction is impaired, ScvO_2_ is unhelpful because it often remains normal. Also, during shock, when oxygen delivery is below its critical level, any increase in it is mainly used for increasing the oxygen consumption, such that ScvO_2_ does not increase as much as would be expected from the increase in CO.

## Conclusions

Along with recommendations [9], it is reasonable today to defend CO monitoring in patients with shock. Of course, it should be reserved for the most critical cases, when initial treatment is ineffective [9]. For sure, all techniques are not equally accurate or invasive and do not provide the same amount of information; the choice should depend on the context and clinicians’ experience. Beyond CO, monitoring devices provide information that helps for diagnosis and management. The invasiveness of transpulmonary or classic thermodilution is not acceptable during routine surgical interventions, but their complication rate is compatible with the severity of critically ill patients [10]. Moreover, clinicians should be taught on how to use these techniques and should be aware of their limitations. Such teaching might also help clinicians understand the complex physiology of CO and tissue oxygenation. In any case, keeping in mind the arguments in favour of and against CO monitoring might help them make an informed choice.

## Abbreviations

CO: Cardiac output
ScvO_2_: Oxygen saturation of the central venous blood
